# Noninvasive Chromosome Screening for Evaluating the Clinical Outcomes of Patients With Recurrent Pregnancy Loss or Repeated Implantation Failure

**DOI:** 10.3389/fendo.2022.896357

**Published:** 2022-06-20

**Authors:** Haitao Xi, Lin Qiu, Yaxin Yao, Lanzi Luo, Liucai Sui, Yanghua Fu, Qiuyi Weng, Jing Wang, Junzhao Zhao, Yingzheng Zhao

**Affiliations:** ^1^ Department of Pharmaceutics, School of Pharmaceutical Sciences, Wenzhou Medical University, Wenzhou, China; ^2^ Department of Obstetrics and Gynecology, Reproductive Medicine Center, The Second Affiliated Hospital of Wenzhou Medical University, Wenzhou, China; ^3^ Department of Clinical Research, Yikon Genomics, Suzhou, China

**Keywords:** noninvasive chromosome screening, repeated implantation failure, recurrent pregnancy loss, assisted reproductive technology, clinical outcomes

## Abstract

This retrospective cohort study explores whether noninvasive chromosome screening (NICS) for aneuploidy can improve the clinical outcomes of patients with recurrent pregnancy loss (RPL) or repeated implantation failure (RIF) in assisted reproductive technology. A total of 273 women with a history of RPL or RIF between 2018 and 2021 were included in this study. We collected data of all oocyte retrieval cycles and single blastocyst resuscitation transfer cycles. For the patients experiencing RPL, NICS reduced the miscarriages rate per frozen embryo transfer (FET), improved the ongoing pregnancies rate and live birth rate: 17.9% vs 42.6%, adjusted OR 0.39, 95% CI 0.16–0.95; 40.7% vs 25.0%, adjusted OR 2.00, 95% CI 1.04–3.82; 38.9% vs 20.6%, adjusted OR 2.53, 95% CI 1.28–5.02, respectively. For the patients experiencing RIF, the pregnancy rates per FET in the NICS group were significantly higher than those in the non-NICS group (46.9% vs. 28.7%, adjusted OR 2.82, 95% CI 1.20–6.66). This study demonstrated that the selection of euploid embryos through NICS can reduce the miscarriage rate of patients experiencing RPL and improve the clinical pregnancy rate of patients experiencing RIF. Our data suggested NICS could be considered as a possibly useful screening test in clinical practice.

## Introduction

*In vitro* fertilization-embryo transfer (IVF-ET) is an effective method to treat infertility widely performed worldwide (1). Nevertheless, chromosomal abnormalities often exist in early human embryos, leading to embryo implantation failure and pregnancy loss during IVF treatment (2, 3), especially in patients with a history of recurrent pregnancy loss (RPL) and repeated implantation failure (RIF) (4–6). RPL is defined as the loss of ≥2 pregnancies, which is confirmed at least by either serum or urine b-hCG, i.e. including non-visualized pregnancy losses (biochemical pregnancy losses and/or resolved and treated pregnancies of unknown location) (7), whereas RIF is the failure of ≥3 implantations (8). Sahoo et al. (9) reported that chromosome abnormalities were detected in 3,975 of 7,396 (53.7%) cases of miscarriage tissues. Besides, Kort et al. (6) analyzed 10,711 cases of blastocysts retrospectively and found that the incidence of embryo aneuploidy was significantly higher in patients experiencing RIF than in the control group.

Currently, the most commonly used embryo selection method is morphological evaluation. However, the chromosomes of embryos cannot be identified *via* morphology (2, 3). Among blastocysts with good morphology, only 42% of embryos had normal chromosomes. Among these, only 30% of ICM graded A Embryos, the chromosomes, were normal (10). Therefore, in some special populations [such as women with recurrent miscarriage and repeated implantation failure (RIF)], embryos are usually evaluated through pre-implantation genetic testing for aneuploidy (PGT-A) (11–13). A retrospective study showed that the live birth and clinical pregnancy rate were improved through the PGT-A in women with RPL (14). The authors included 1,389 blastocysts derived from PGT-A cycles in IVF patients with advanced maternal age, those with RIF, those with recurrent miscarriage, and oocyte donors. Compared to that in the control group without PGT-A, the live birth rates of the four groups were improved (15). However, specific equipment and extensive expertise are required for the biopsy procedure, restraining the utility of PGT-A in assisted reproduction. Moreover, the embryos were screened for chromosomal ploidy before transferring to the uterus (16, 17), and the long-term impact of biopsy is an important concern for undetermined health risks, such as adrenal development and response to cold stress (18), epigenetic reprogramming (19), and neurological conditions (20). Notably, implantation rates were lower with increased biopsied trophectoderm (TE) cell numbers and sizes than with appropriate cell numbers and sizes (21, 22).

Stigliani et al. (23) first observed genomic DNA contents in embryo culture medium. Since then, multiple studies have been published using culture medium or blastocoelic fluid for analyzing chromosomal ploidy (24–31). Xu et al. (27) first reported a noninvasive chromosome screening (NICS) assay based on a Multiple Annealing and Looping-Based Amplification Cycle-Next Generation Sequencing (NGS) strategy using spent blastocyst culture medium, which was validated in 42 IVF and resulted in five live births among seven women. Fang et al. (29) obtained an ongoing pregnancy rate of 58% and reported 27 normal live births in a pilot clinical study using NICS. Nevertheless, the clinical application of NICS has been evaluated only in small-scale trials.

Here, we designed a retrospective cohort study including 273 patients experiencing RPL or RIF to confirm the clinical value of NICS. To the best of our knowledge, this is the first large-scale validation study of NICS in the patients experiencing RPL or RIF.

## Materials and Methods

### Study Participants and Data Collection

We initially included 303 women with a history of RPL (≥2 pregnancies) (7) or RIF (≥3implantations) (8), exclusion criteria were APS, diabetes, hypothyroidism or other severe complications, from July 2018 to May 2021, according to the records of the Reproductive Centre at the Second Affiliated Hospital of Wenzhou Medical University. However, 10 women that abandoned embryo transfer and 20 women with chromosomal rearrangements, abnormal uterine cavity morphology, endometrial lesions, endometrial injury, intrauterine effusion, or untreated hydrosalpinx were excluded from the study. The final cohort comprised 273 women, from which we collected data of all oocyte retrieval cycles and single blastocyst resuscitation transfer cycles.

**Figure 1:**
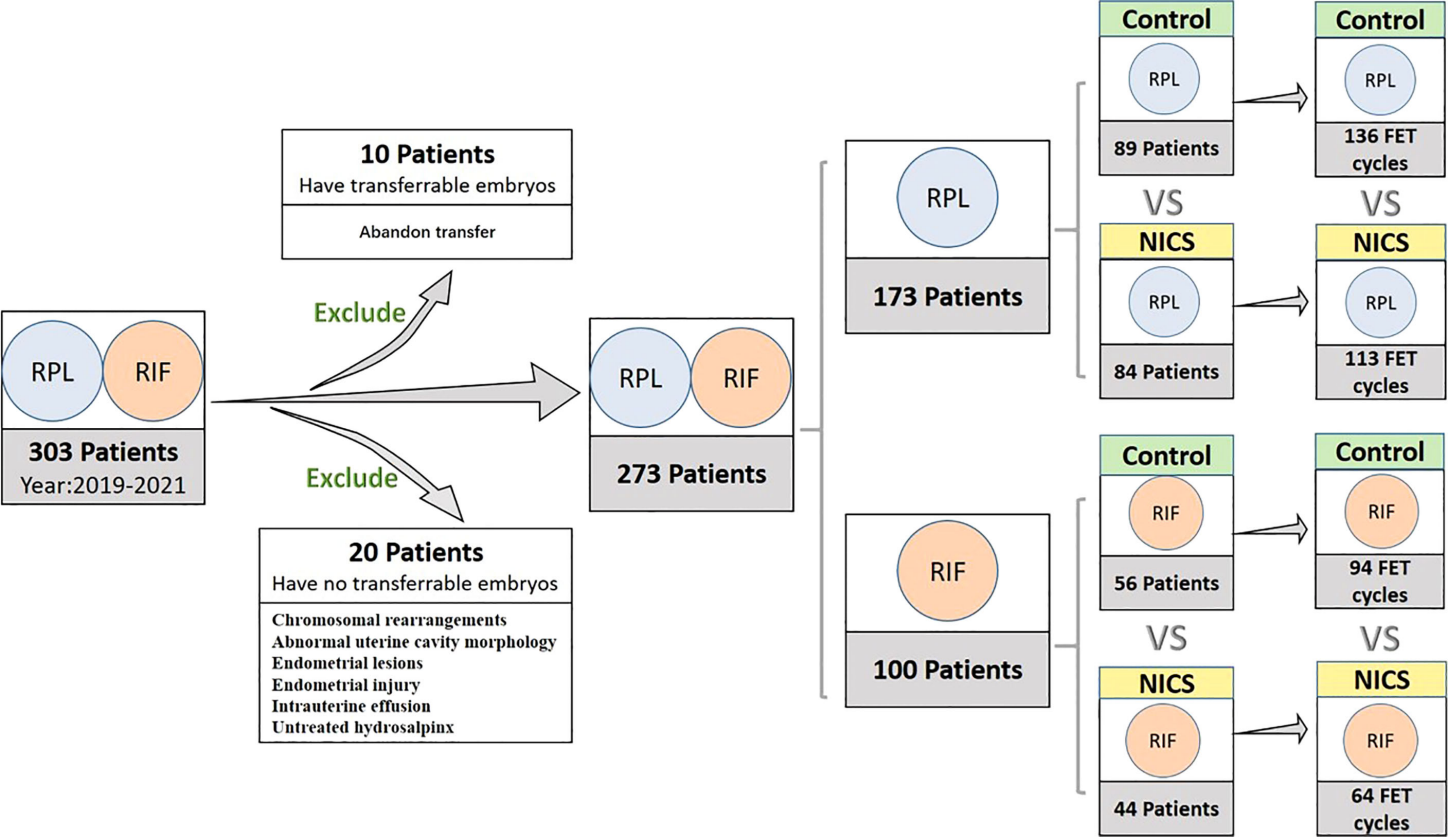
Inclusion and classification of patient selection in this study. NICS, noninvasive chromosome screening; RPL, recurrent pregnancy loss; RIF, repeated implantation failure; Control, blastocyst morphology (non-NICS).

The variables analyzed in the study were: age at retrieval, the history of pregnancies, live births, and miscarriages, body mass index (BMI), hormone levels, the number of oocyte retrieval cycles, oocytes, cleavages, and D5 or D6 blastocysts, and blastocyst morphology (expansion, inner cell mass, and trophectoderm). After consulting patients, we divided them into two groups. Patients experiencing RPL or RIF who received the noninvasive chromosome screening for aneuploidy were included in the NICS group, while those who underwent conventional morphology embryo transfer during the same period were included in the non-NICS group. The euploid embryos were transferred to the NICS group. The study design is illustrated in **Figure 1**.

### Oocyte Retrieval and Embryo Culture

This study was performed at the first IVF/Intracytoplasmic Sperm Injection (ICSI) cycles after injection of 3.75 mg triptorelin for prolonged pituitary downregulation in the follicular phase of the menstrual cycle. Ovarian stimulation with exogenous gonadotropins promoted the growth of follicles. When two or more leading follicles reached 18 mm, ovulation was induced with 10,000 IU human chorionic gonadotropin (hCG). Oocyte retrieval was performed at 35 h post-hCG administration. Cumulus-enclosed oocytes were separated from the follicular fluid, placed in a medium, and incubated at 37°C incubated d Cumulus-encl_2_ atmosphere for 2 h. Routine IVF or ICSI was performed based on sperm quality. The embryos were placed in droplets of G-1 PLUS medium (Vitrolife, Göteborg Sweden) in AMP-30D incubators (Bioz, Los Altos, CA, USA) in a 6.0% CO_2_ and 5% O_2_ balance N_2_ atmosphere at 37°C.

### Blastocyst Culture and Transfer

D3 embryos were placed in 30-μL droplets of G-2 PLUS medium (Vitrolife) supplemented with washed and pre-gassed mineral oil (Sage, Atlanta, GA, USA) and cultured to the blastocyst stage in AMP-30D incubators (Bioz) in a 6.0% CO_2_ and 5% O_2_ balance N_2_ atmosphere at 37°C. At 2 d of culture, the development and quality of blastocysts were evaluated according to the blastocyst scoring system, including expansion (1–6), inner cell mass (A, B, C), and trophectoderm (A, B, C). Blastocysts were categorized into good, fair, or poor quality based on the simplified SART embryo scoring system (30). The grade is good when the ICM/TE is AA, AB, or BA; the grade is fair when the ICM/TE is BB or AC; the grade is poor when the ICM/TE is CA, BC, or CB. All blastocysts were previously frozen and thawed by vitrification using the Cryotop Safety kit (Kitazato, Fuji-shi, Shizuoka, Japan), according to the manufacturer’s instructions. A single blastocyst was selected for transfer to each patient based on morphology (nonintervention group) or NICS results (intervention group).

### Sample Collection for NICS Assay

To prevent medium cross-contamination, different Pasteur pipettes were used for each embryo. Approximately 30 μL of blastocyst medium from each embryo was transferred into RNase-DNase-free PCR tubes containing 5 μL of cell lysis buffer (Yikon Genomics, Suzhou, China). the same amount of blastocyst culture medium was collected to be used as a negative control. All collected samples were flash-frozen in liquid nitrogen and stored at -80°C until subjected to the NICS assay.

### Whole-Genome Amplification and NGS Data Analysis

Whole-genome amplification was performed using culture media, followed by library preparation using ChromInst (Yikon Genomics; EK100100724 NICS Inst Library Preparation Kit). NGS was performed on an Illumina MiSeq platform that yielded approximately 2 million sequence reads from each sample. The high-quality reads were extracted and mapped to the human hg19 genome. After removing duplication reads, the read numbers were counted along the whole genome with a bin size of 1 Mb and normalized by the GC content, as well as a reference dataset to represent the relative copy number. The copy number of each bin was then segmented by circular binary segmentation (CBS) algorithms to merge bins with similar trends and calculate the final copy numbers.

### Assessment of Outcome Variables

Our primary outcomes included the clinical pregnancy rates, miscarriage rates, and ongoing pregnancy rates. The clinical pregnancy rate was defined as the number of cycles with gestational sacs visualized on transvaginal ultrasonography divided by the total number of transplant cycles. The miscarriage rate was calculated as the number of pregnancy failures after a gestational sac was documented by transvaginal ultrasonography divided by the total number of clinical pregnancies. The ongoing pregnancy rate was the number of cycles of any pregnancy that went beyond 12 weeks of gestation, divided by the total number of transplant cycles. Ectopic pregnancy was considered a clinical pregnancy and not a miscarriage.

### Statistical Analysis

Descriptive data for continuous variables are presented as means ± standard deviation, whereas categoric data as numbers and percentages. The Student’s *t*-test or Mann–Whitney test were used to identify significant differences in parametric and non-parametric data, respectively. The chi-square test or Fisher exact test were used for categoric variables in each group. Multiple logistic regression analysis was conducted to compare the outcomes of NICS and non-NICS groups after controlling the covariables at *p* < 0.10 and covariables considered clinically influential.

All analyses were conducted using the statistical software BMI SPSS, Version 25. A P-value < 0.05 was considered denoting statistical significance.

### Ethics Approval and Consent to Participate

This study was approved by the Institutional Review Board (IRB) of the Second Affiliated Hospital of Wenzhou Medical University (number: 2021-K-13-01). Informed consent was obtained in accordance with the institutional guidelines before embryo analysis.

## Results

### Variable Analysis

Of the 303 women initially included in the study, 10 that abandoned embryo transfer and 20 that had no transferrable embryos were excluded from the final cohort. Finally, blastocysts were transferred to 273 women during 407 transfer cycles (**Figure 1**).

Of the 173 patients with a history of RPL, were enrolled in the study, of which 84 were for the NICS group and 89 were included as the non-NICS group. In total, 113 FET cycles were performed for the patients in the NICS group, and 136 high-quality blastocysts were obtained from 89 patients (**Table 1**). Of the 100 patients with a history of RIF, 44 were for the NICS group and 56 for the non-NICS group. In total, 64 FET cycles were performed for the patients in the NICS group, and 94 high-quality blastocysts were obtained from the non-NICS group (**Table 2**).

**Table 1:** Baseline demographics of patients experiencing recurrent pregnancy loss (RPL) after embryo selection by noninvasive chromosome screening (NICS) versus blastocyst morphology (Non-NICS).

Variable	NICS	Non-NICS	*p*-value
Number of patients	84	89	
Female age (y)	32.4 ± 3.8	33.4 ± 4.7	0.109
Female body mass index (kg m^-2^)	22.1 ± 2.8	22.4 ± 3.1	0.371
Male age (y)	34.7 ± 4.1	35.2 ± 4.7	0.381
Male body mass index (kg m^-2^)	24.0 ± 3.0	24.5 ± 3.2	0.293
Infertility duration (y)	1.4 ± 0.7	2.1 ± 2.2	0.001
Number of prior miscarriages	2.9 ± 1.3	2.5 ± 1.1	0.023
Number of previous embryo transfers	0.63 ± 1.0	2.2 ± 1.8	<0.001
Number of prior live births	5 (6.0%)	1 (1.1%)	0.108
Prolactin (ng m^-1^)	20.0 ± 44.9	21.4 ± 45.9	0.831
Testosterone	0.5 ± 0.3	0.9 ± 4.3	0.415
Progesterone	0.7 ± 0.5	0.6 ± 0.6	0.357
Follicle-stimulating hormone	6.8 ± 2.1	7.5 ± 4.2	0.158
Luteinizing hormone	4.8 ± 3.7	5.8 ± 9.6	0.349
Estradiol	62.7 ± 58.1	66.7 ± 71.88	0.673
Number of COH cycles	87	105	
Number of COH cycles per patient	1.04	1.18	0.007
Gonadotropin days (x̅ ± SD)	10.2 ± 2.1	10.5 ± 2.9	0.416
Gonadotropin dosage (×75 IU, x̅ ± SD)	2,065.3 ± 768.1	1,956.3 ± 856.9	0.359
Number of retrieval oocytes (x̅ ± SD)	12.8 ± 7.0	12.8 ± 7.3	0.977
Number of cleaving zygotes (x̅ ± SD)	8.9 ± 4.7	9.5 ± 6.0	0.441
Number of blastocysts	4.7 ± 3.3	4.4 ± 3.1	0.513
Number of FET cycles	113	136	
Embryos at day 5	80 (70.8%)	114 (83.8%)	0.014
Embryos at day 6	33 (29.2%)	22 (16.2%)	
Blastocyst quality, Good (AA/BA/AB)	36 (31.9%)	45 (33.1%)	0.837
Blastocyst quality, Fair (BB/AC)	35 (31.0%)	58 (42.6%)	0.058
Blastocyst quality, Poor (CA/BC/CB)	42 (37.2%)	33 (24.3%)	0.027
Number of embryos	371		
Aneuploidy rate	195/371(52.56%)		

COH, controlled ovarian hyperstimulation; SD, standard deviation; FET, frozen-thawed embryo transfer. A total of 371 embryos were examined in the RPL group for aneuploidy.

**Table 2:** Baseline demographics of patients experiencing repeated implantation failure (RIF) after embryo selection by noninvasive chromosome screening (NICS) versus blastocyst morphology (Non-NICS).

Variable	NICS	Non-NICS	*p*-value
Number of patients	44	56	
Female age (y)	32.1 ± 4.7	33.3 ± 4.3	0.143
Female body mass index (kg m^-2^)	21.0 ± 2.5	21.7 ± 2.4	0.101
Male age (y)	34.2 ± 5.2	34.9 ± 4.9	0.407
Male body mass index (kg m^-2^)	24.2 ± 3.4	23.8 ± 3.4	0.498
Infertility duration (y)	3.5 ± 2.9	5.2 ± 3.0	0.004
Number of prior miscarriages	1.9 ± 1.2	0.8 ± 1.1	<0.001
Number of previous embryo transfers	4.4 ± 1.3	4.2 ± 1.3	0.379
Number of prior live births	1/44 (2.3%)	0	
Prolactin (ng m^-1^)	26.2 ± 76.4	15.1 ± 9.9	0.231
Testosterone	0.5 ± 0.2	0.5 ± 0.3	0.973
Progesterone	0.8 ± 1.0	0.6 ± 0.3	0.287
Follicle-stimulating hormone	7.8 ± 2.4	7.5 ± 3.5	0.608
Luteinizing hormone	5.2 ± 2.2	4.5 ± 2.7	0.108
Estradiol	50.4 ± 25.8	51.2 ± 41.70	0.913
Number of COH cycles	44	70	
Number of COH cycles per patient	1	1.3	<0.001
Gonadotropin days (x̅ ± SD)	10.0 ± 2.4	11.0 ± 2.4	0.03
Gonadotropin dosage (×75 IU, x̅ ± SD)	1,838.4 ± 748.3	2,192.1 ± 872.7	0.028
Number of retrieval oocytes (x̅ ± SD)	13.9 ± 7.5	13.0 ± 7.5	0.551
Number of cleaving zygotes (x̅ ± SD)	10.8 ± 6.1	9.7 ± 5.4	0.319
Number of blastocysts (x̅ ± SD)	5.3 ± 2.8	4.2 ± 3.2	0.059
Number of FET cycles	64	94	
Embryos at day 5	48 (75.0%)	64 (68.1%)	0.24
Embryos at day 6	16 (25.0%)	30 (31.9%)	
Blastocyst quality, Good (AA/BA/AB)	11 (17.2%)	22 (23.4%)	0.345 0.124
Blastocyst quality, Fair (BB/AC)	31 (48.4%)	34 (36.2%)	
Blastocyst quality, Poor (CA/BC/CB)	22 (34.4%)	38 (40.4%)	0.442
Number of embryos	189		
Aneuploidy rate	107/189 (56.61%)		

COH, controlled ovarian hyperstimulation; SD, standard deviation; FET, frozen-thawed embryo transfer. A total of 189 embryos were examined in the RIF group for aneuploidy.

Data variables analyzed in this study were age at retrieval, the history of pregnancies, live births, and miscarriages, BMI, hormone levels, the number of oocyte retrieval cycles, oocytes, cleavages, and D5 or D6 blastocysts, and blastocyst morphology (expansion, inner cell mass, and trophectoderm) were comparable by Student’s t-test or Mann–Whitney *U* test, each as appropriate (**Tables 1**, **2**).

Our analysis showed no significant differences in age, BMI, and hormone levels between the NICS and non-NICS groups in patients experiencing RPL or RIF. The number of previous miscarriages in the NICS group of patients experiencing RPL or RIF was significantly higher than in the non-NICS group. Besides, infertility duration showed statistical significance (*p =* 0.001, *p =* 0.004) in the NICS group and non-NICS group, no matter which feature the patients were in. The number of miscarriages and infertility duration affected clinical outcomes. Thus, they were included in the multiple logistic regression analysis.

### Blastocyst Morphological Assessments and NICS Results

The blastocyst transplantation was based on morphology and NICS results. Blastocysts were evaluated based on their development and quality using the Gardner scores system and divided into three categories: good (AA/BA/AB), fair (BB/AC), and poor (CA/BC/CB) (32). As shown in **Tables 1**, **2**, no significant differences were observed between the NICS group and the control group of patients experiencing RIF. Of the patients experiencing RPL, the non-NICS group was lower than the NICS group (37.2% vs. 24.3%) in the poor category; however, there was no significant difference between the other two categories.

In the NICS assay, we sequenced approximately 2 million reads on each culture medium sample. The read numbers were counted along the 24 chromosomes with a bin size of 1 Mb and normalized by the mean of the corresponding bin in all samples. Examples of the results of NICS were demonstrated in **Additional File 1**.

### Comparison of Clinical Outcomes Between NICS and Non-NICS Groups for Patients Experiencing RPL or RIF

**Figure 2:**
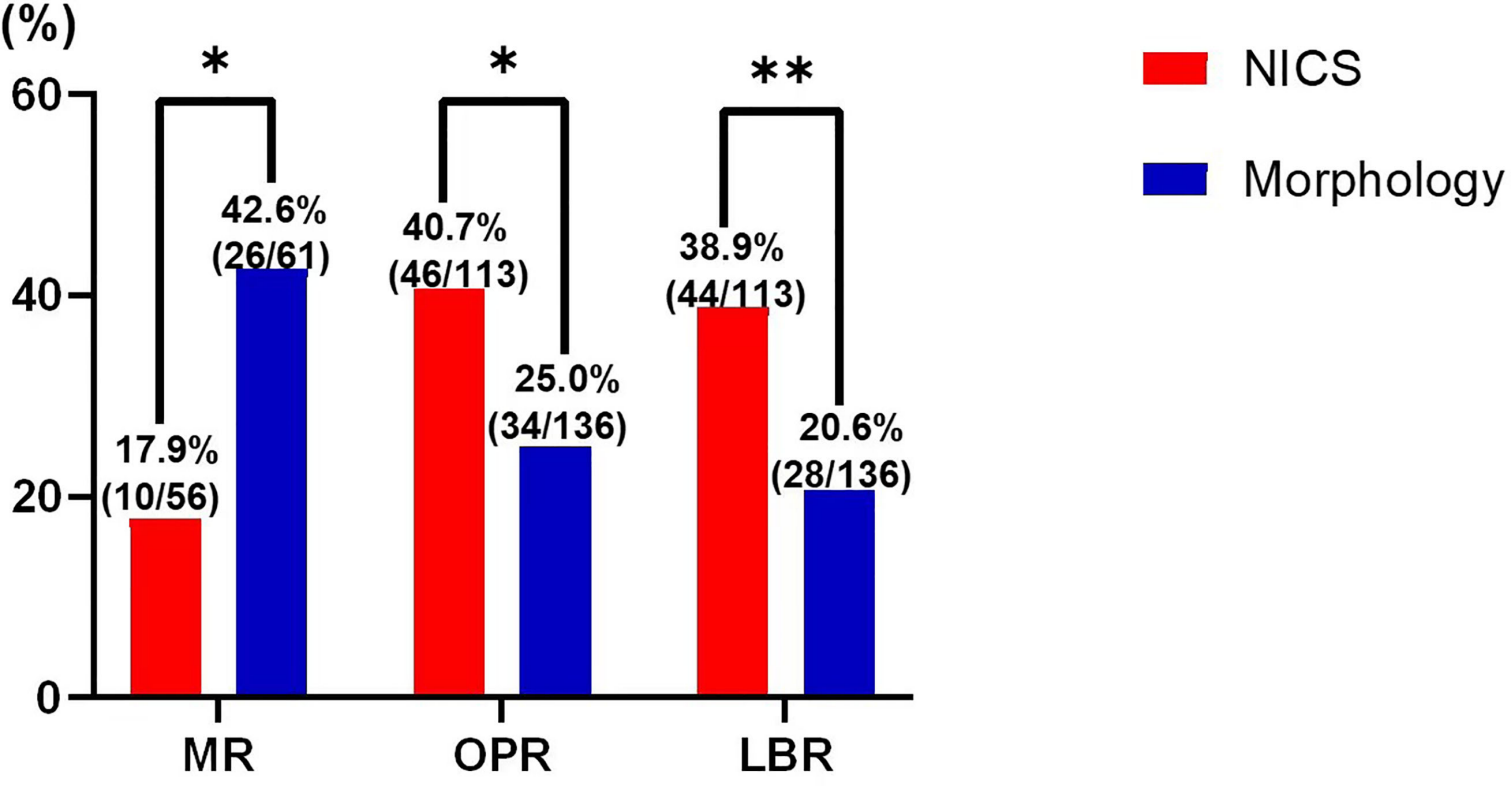
Pregnancy outcomes of patients with recurrent pregnancy loss (RPR) after embryo selection by noninvasive chromosome screening (NICS) versus blastocyst morphology (control). MR, miscarriages rate; OPR, ongoing pregnancy rate; LBR, live birth rate. *p < 0.05, **p < 0.01.

For the patients experiencing RPL, the miscarriage rate per FET was significantly lower in the NICS group than in the non-NICS group (17.9% vs. 42.6%, adjusted OR 0.39, 95% CI 0.16–0.95), whereas the ongoing pregnancy rate (40.7% vs. 25.0%, adjusted OR 2.0, 95% CI 1.04–3.82) and live birth rate (38.9% vs. 20.6%, adjusted OR 2.53, 95% CI 1.28–5.02) were significantly higher in the NICS group compared to the non-NICS group (**Figure 2**; **Table 3**). Nevertheless, no differences were identified in pregnancy rates per patient between the NICS and non-NICS groups (49.6% vs. 44.9%, adjusted OR 1.13, 95% CI 0.61–2.07; **Table 3**).

**Table 3:** Comparison of clinical outcomes patients experiencing recurrent pregnancy loss (RPL).

	NICS	Non-NICS	Adjusted odds ratio (95% CI), *p*-value
Number of transferred cycles	113	136	
Clinical pregnancies rate (%)	56/113 (49.6%)	61/136 (44.9%)	1.13 (0.61–2.07), 0.697
Miscarriages rate (%)	10/56 (17.9%)	26/61 (42.6%)	0.39 (0.16–0.95), 0.038
Ectopic pregnancies	0	1/61 (1.6%)	
Ongoing pregnancies rate (%)	46/113 (40.7%)	34/136 (25.0%)	2.0 (1.04–3.82), 0.037
Live birth rate (%)	44/113 (38.9%)	28/136 (20.6%)	2.53 (1.28–5.02), 0.008

NICS group, embryo selection by noninvasive chromosome screening; control group, blastocyst morphology.

*Adjusted for female age, infertility duration, number of prior miscarriages, number of previous embryo transfers, number of COH cycles, embryo morphology, and Day 5/6 blastocysts.

**Figure 3:**
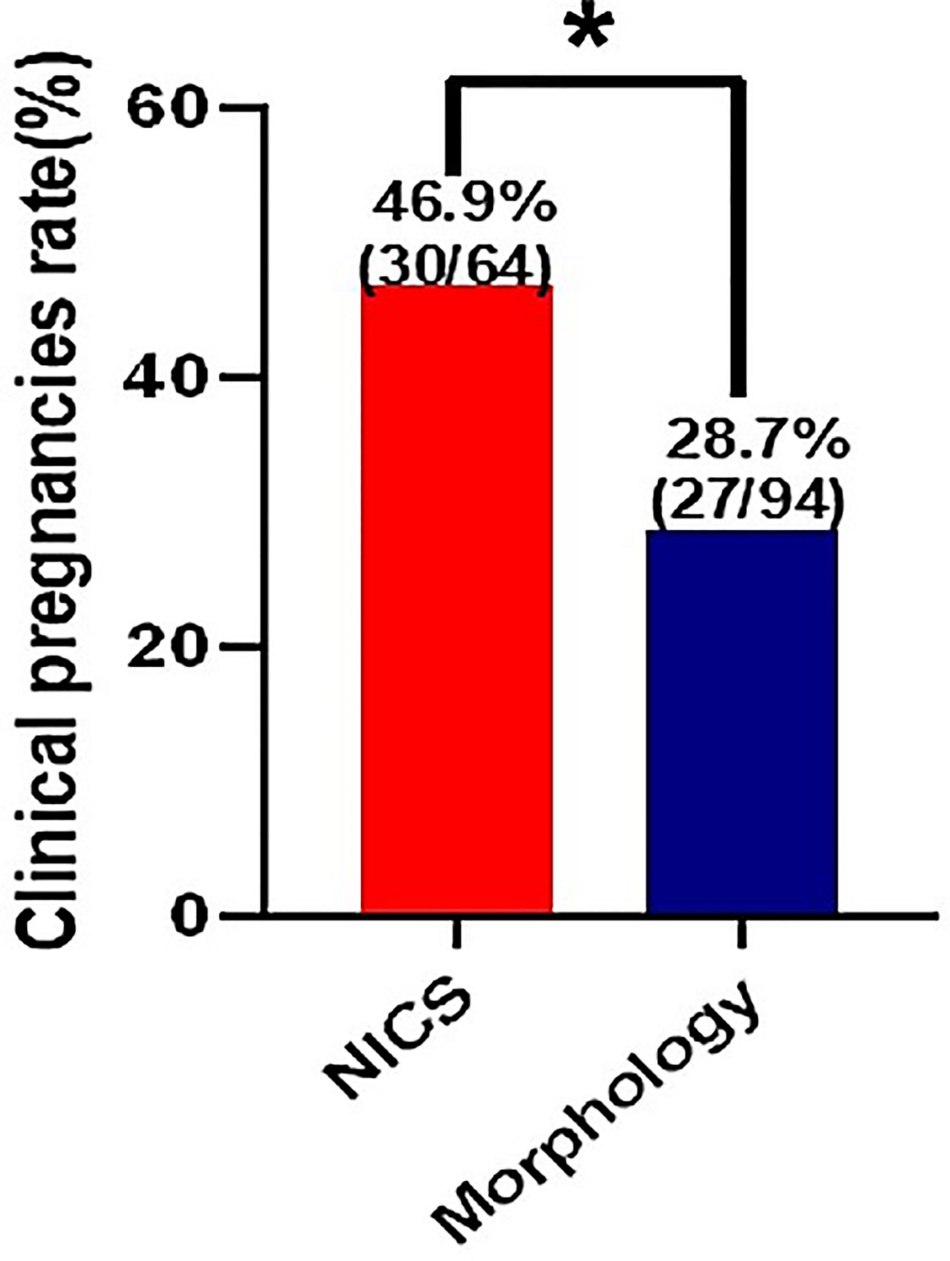
Clinical pregnancy rate of patients with repeated implantation failure (RIF) after embryo selection by noninvasive chromosome screening (NICS) versus blastocyst morphology (control). *p < 0.05.

For the patients experiencing RIF, the pregnancy rates per FET were significantly higher in the NICS group than in the non-NICS group (46.9% vs. 28.7%, adjusted OR 2.82 95% CI 1.20–6.66. **Figure 3**; **Table 4**), whereas the live birth rate and ongoing pregnancy rate per FET and per patient were no significant difference in the NICS group than in the non-NICS group. Nevertheless, no differences were identified in the miscarriage rate per clinical pregnancy between the NICS and non-NICS groups (23.3% vs. 25.9%, adjusted OR 1.35, 95% CI 0.34–5.42; **Table 4**).

**Table 4:** Comparison of clinical outcomes patients experiencing recurrent implantation failure.

	NICS	Non-NICS	Adjusted odds ratio (95% CI), *p*-value	
Number of transferred cycles	64	94	
Clinical pregnancies rate (%)	30/64 (46.9%)	27/94 (28.7%)	2.82 (1.20–6.66), 0.018
Miscarriages rate (%)	7/30 (23.3%)	7/27 (25.9%)	1.35 (0.34–5.42), 0.671
Ectopic pregnancies	3/30 (10%)	0	
Ongoing pregnancies rate (%)	20/64 (31.3%)	20/94 (21.3%)	2.19 (0.88–5.47), 0.094
Live birth rate (%)	19/64 (29.7%)	20/94 (21.3%)	1.96 (0.78–4.92), 0.154

NICS group, embryo selection by noninvasive chromosome screening; control group, blastocyst morphology.

*Adjusted for female age, infertility duration, number of prior miscarriages, number of COH cycles, gonadotropin days, gonadotropin dosage, number of blastocysts embryo morphology and Day 5/6 blastocysts.

## Discussion

To our knowledge, this is the first large-scale study to confirm the effectiveness of NICS as a diagnostic test for the outcomes of IVF in women with a history of RPL or RIF. We demonstrated that the miscarriage rate was significantly lower in women with RPL (n = 173), whereas the pregnancy rate was significantly higher in the NICS group than in the non-NICS group of women with RIF (n = 100). To confirm that the improvement of clinical outcomes in the NICS group was related to the genetic testing alone despite the other factors, we included the variables at *p <* 0.10 and related to the clinical outcome in the adjusted models. Our results strongly supported the high clinical value of NICS in women with RPL before embryo transfer since it significantly increased the live birth rate per transfer cycle through the multiple logistic regression analysis (38.9% vs. 20.6%, adjusted OR 2.53, 95% CI 1.28-5.02). The pregnancy rates per FET in the NICS group were significantly higher than in the non-NICS control group (46.9% vs. 28.7%, adjusted OR 2.82, 95% CI 1.20–6.66) for the patients experiencing RIF. Similarly, Fang et al. obtained an ongoing pregnancy rate of 58% and reported 27 normal live births in patients experiencing RPL or RIF after transferring 50 embryos identified as euploid by NICS (29).

In this study, women with RPL had a history of ≥2 miscarriages, spontaneous abortion, and/or biochemical pregnancy, as described in the ESHRE guideline (7, 31). The definition of RPL differs among organizations. For instance, the Royal College of Obstetricians and Gynecologists and the European Society of Human Reproduction and Embryology support that a miscarriage includes all pregnancy ≥3 losses up to 24 weeks of gestation, whereas the American Society for Reproductive Medicine does not provide a time limit and ≥2 miscarriages of pregnancy, excludes biochemical pregnancy (32). The rate of chromosome abnormality decreases with the developmental stage: 70–80% in clinical miscarriages (33), 4% in stillbirths, and 0.3% in newborn babies (34). The selection of euploid embryos can reduce the rate of miscarriages, which are probably caused by chromosome abnormalities (35).

Women with RIF had a history of ≥3 failed implantations of 4–6 high-score blastomeres or >3 high-score blastocysts (8). A retrospective analysis of 10,711 blastocysts showed that the incidence of blastocyst aneuploidy in patients experiencing RIF is significantly higher than that in the control group, and embryo aneuploidy is a primary reason for implantation failure (6). A clinical study suggested that transplanting euploid embryos may help patients experiencing RIF to achieve pregnancy (36). A multi-center, prospective, pilot study showed that screening for embryo ploidy improved the live birth rate per embryo transfer procedure in both RPL (52.4% vs 21.6%) and RIF group (62.5% vs 31.7%), which indicated PGT-A has an advantage reducing the number of embryo transfer cycles (37).

Chromosome aneuploidy is common in embryos following IVF, even in younger women, and is a major failure factor. Although morphology is correlated with euploidy, it can only increase euploidy by a few percentage points at most when used to select replacement embryos. Embryos with good morphology may have chromosomal abnormalities (10). Routine embryo screening for aneuploidy requires intensive biopsy procedures, which are costly and time-consuming. The accuracy of NICS has been verified in many studies, and a 58% clinical pregnancy success rate has been reported for patients experiencing RIF and RPL (29). Recently, the utility of niPGT-A is challenged in the article by Hanson et al. (38). Here, we also found that NICS allowed the selection of embryos, increasing the live birth rates in women with RPL and improving the clinical pregnancy rates in women with RIF based on many patients, which is meaningful to both clinicians and basic scientists on NICS assay. Chen et al. (39) assessed the performance by comparing with the CNV from the whole embryo assay as the gold standard. The differences of sensitivity, specificity, NPV, and PPV between TE-PGT and NICS were not statistically significant. Our center is participating in a multi-unit clinical trial for women ≥35 years of age to further validate the clinical value of NICS since a more comprehensive application requires advanced technology such as modified WGA and sequencing protocols and novel diagnostic algorithms. The validation of NICS in different populations and the accumulation of detectable sample sizes may help markedly reduce the detection cost per embryo.

There are limitations in our study: 1) as a retrospective study, patients in the NICS groups had different clinical prognoses than those in the non-NICS groups, introducing bias in the study results, and 2) the loss and cycle cancelation caused by euploid selection after NICS was not calculated since only women with a good prognosis reach the blastocyst stage and have an euploid embryo to transfer.

Our large-scale retrospective study demonstrated noninvasive chromosome screening for aneuploidy improved the clinical outcomes for patients experiencing RPL or RIF. NICS could be considered as a possibly useful screening test in clinical practice.

## Data Availability Statement

Data of this project can be accessed after an approval application to the China National Genebank (CNGB, https://db.cngb.org/cnsa/). Please refer to https://db.cngb.org/ for detailed data, accession number CNP0002817.

## Ethics Statement

The studies involving human participants were reviewed and approved by the Institutional Review Board (IRB) of the Second Affiliated Hospital of Wenzhou Medical University. The patients/participants provided their written informed consent to participate in this study.

## Author Contributions

HX conceived of the presented idea and the experiments, developed the theory, performed the computations and wrote manuscript; LQ developed the theory, analyzed data and wrote manuscript; YY and LL carried out the experiment and investigation; LS helped to perform the analysis with constructive discussions.; YF and QW collected and analyzed data; JW assisted with the experiments; JZ and YZ administrated the whole project. All authors discussed the results and contributed to the final manuscript.

## Funding

This study was supported by the Research Fund for Lin He’s Academician Workstation of New Medicine and Clinical Translation at the Second Affiliated Hospital of Wenzhou Medical University (grant no. 18331105) and the National Key Research and Development Program (grant no. 2018YFC1003103).

## Conflict of Interest

The authors declare that the research was conducted in the absence of any commercial or financial relationships that could be construed as a potential conflict of interest.

## Publisher’s Note

All claims expressed in this article are solely those of the authors and do not necessarily represent those of their affiliated organizations, or those of the publisher, the editors and the reviewers. Any product that may be evaluated in this article, or claim that may be made by its manufacturer, is not guaranteed or endorsed by the publisher.
