# Supplementary figures and images for: Noninvasive Chromosome Screening for Evaluating the Clinical Outcomes of Patients With Recurrent Pregnancy Loss or Repeated Implantation Failure
Source: Front Endocrinol (Lausanne). 2022 Jun 20;13:896357. doi: 10.3389/fendo.2022.896357 (PMC9253989; doi:10.3389/fendo.2022.896357)

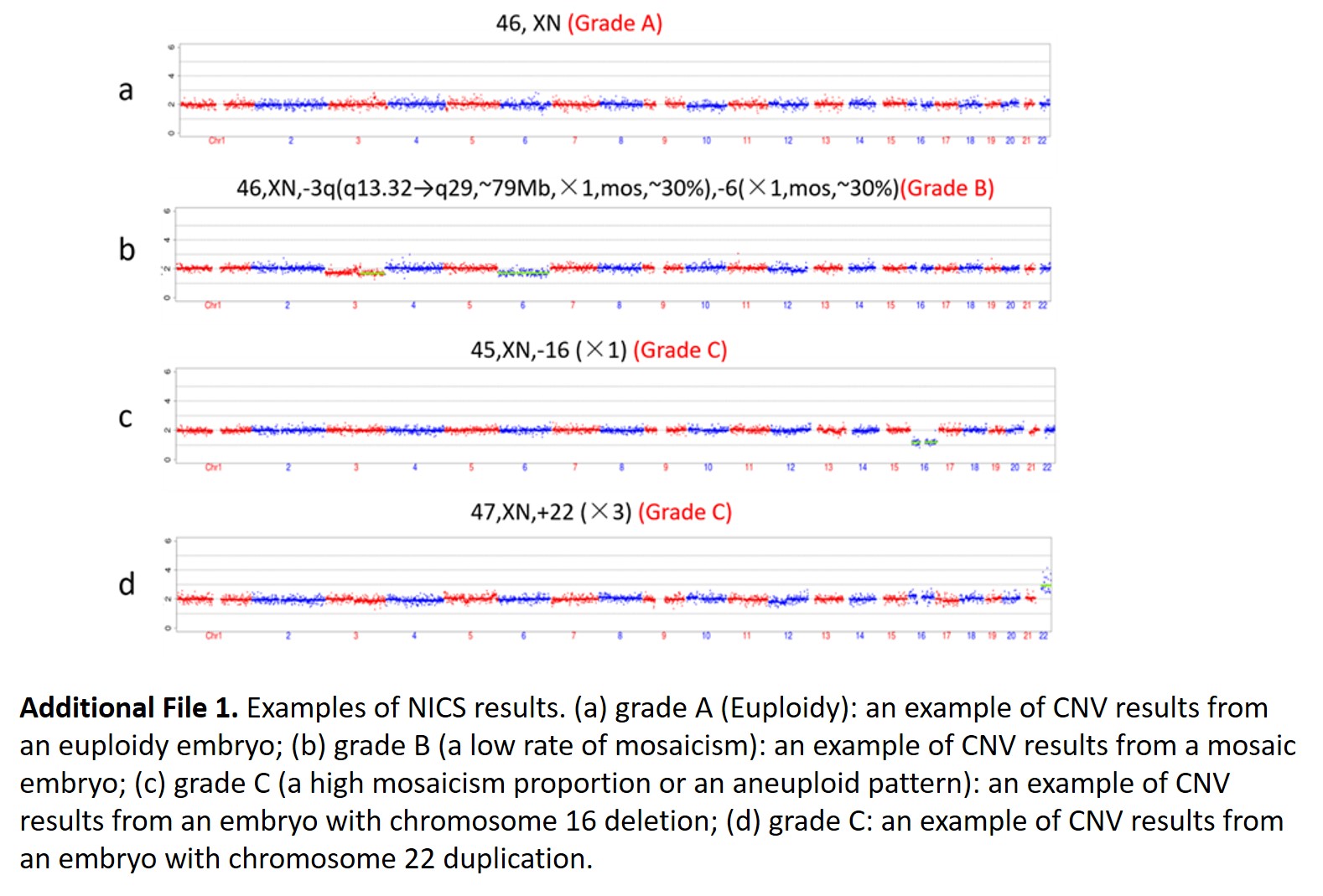

Supplement: Supplementary file 1 [file Image_1.jpeg]
